# Gender, Police Culture, and Structured Ambivalence: Navigating ‘Fit' with the Brotherhood, Boys’ Club, and Sisterhood

**DOI:** 10.1177/15570851221098040

**Published:** 2022-05-03

**Authors:** Carrie B. Sanders, Julie Gouweloos, Debra Langan

**Affiliations:** 1 8431 Wilfrid Laurier University, Brantford, ON, Canada; 2 McMaster University, Hamilton, ON, Canada

**Keywords:** policing, gender, culture, brotherhood, boys’ club, sisterhood

## Abstract

Women are increasingly represented in policing; however, inclusion alone will not eradicate existing structural and cultural barriers to meaningful change. Insights from interviews with ninety-one Canadian women police of varied rank and tenure, demonstrate women’s experiences of structured ambivalence as they strategically deploy and resist gendered policing narratives of the Brotherhood, Boys’ Club, and Sisterhood to negotiate their own ‘fit.’ In this way, they both challenge and reinforce gendered boundaries that create barriers to meaningful transformation. These findings demonstrate the need for change initiatives to address the complex and ever-shifting role of gender in policing organizations.

The representation of women in policing continues to increase across the globe with some police services having between 25% and 30% female officers, and between 30% and 50% female recruits (Prenzler, 2020). However, this progress has been ‘extremely patchy’ with most women holding patrol level positions and a much smaller number entering specialized units (e.g., Guns and Gangs, Homicide), management, and senior management positions (Prenzler, 2020, p. 439). In Canada, women comprise 22.2% of the total police population with less than 19% of senior officer positions^1^ being held by women (Conor et al., 2020). While numbers have been increasing, we caution against equating the slow rise in women’s presence in policing with gender equity. In fact, we contend that simply increasing the number of women in policing will not, in and of itself, create meaningful organizational change. Our findings theorize the structured ambivalence women express toward gender in policing. Specifically, women draw on and resist cultural resources of the Brotherhood, Boys’ Club, and Sisterhood to make sense of and navigate their fit within the hierarchical structure of policing. They do so in ways that both challenge and reinforce the gendered boundaries that create barriers to change. As such, we argue that for meaningful cultural change to be achieved, organizations must account for women’s complex and ever-shifting navigation of gender in police organizations.

Within the literature there is a long-standing recognition that policing is a ‘masculine culture’ (Manning, 1978), one that is gendered at individual, cultural, and structural levels (Silvestri, 2017). However, more recent work has proffered the need to rethink overbroad conceptions of police culture (see Campeau, 2015; Silvestri, 2017). Specifically, Campeau (2015) argues against the perception of police culture as being either a monolithic set of values or individualized typologies (e.g., street cop or management cop culture), and instead regards police culture as a “repertoire of skills that are deployed in order to bring justification to their experiences” (p. 669). We take up Campeau’s (2015) approach to police culture as a “resourceful tool” paying specific attention to understanding *how* women use cultural resources, including the Brotherhood, Boys’ Club, and Sisterhood, to make sense of their experiences and navigate their ‘fit’ (Rabe-Hemp, 2009) within the hierarchical institution of policing.

The existence of a Boys’ Club–the entrenched gendered hierarchy within policing that often excludes women from inclusion, promotion, and full participation–has been widely documented in the policing literature (see Campeau, 2019; Fielding, 1994; Langan et al., 2019; Rabe-Hemp, 2008; 2009). The Brotherhood also plays a prominent role in policing literature. Often used to connote solidarity, the ‘thin blue line,’ or a sense of family, the Brotherhood is a core constituent of police culture. Perhaps unsurprisingly, far less has been said about the existence of a Sisterhood–a shared sense of solidarity among women.^2^ Our work pushes policing culture theory in new directions by unpacking the complex and at times contradictory ways in which women strategically navigate these three prominent cultural resources. Specifically, we ask, *how do women understand, deploy, and resist conceptions of the Brotherhood, Boys’ Club, and Sisterhood to negotiate their own fit within their policing cultures*?

## Gender, Culture, Policing, and Fit: A Review of the Literature

The scholarship on women and policing has provided valuable insights into the gendered reality of police work. Women are promoted less frequently, experience lower retention rates, and are far more likely than men to experience hostile work environments (Strategic Human Resources Analysis of Public Policing in Canada, 2000, p. 74; see also Brown & Silvestri, 2020; Chan et al., 2010; Yu, 2020). In Canada there have been several class action lawsuits and human rights complaints by women police (Human Resources Development Canada, 2000) against their police services that have created heightened public visibility and calls for accountability in the media (Grant, 2020; McQuigg, 2019; Trinh & Baksh, 2021). There is no shortage of evidence indicating that police work is deeply gendered, and researchers have made valuable contributions to our collective understanding of how and why this kind of inequality persists.

Shelley et al. (2011) argue that policing is gendered in the way it legitimizes hegemonic masculinity and filters women into less ‘valuable’ work. Silvestri (2018) encourages us to critically examine how the seemingly neutral concept of ‘time’ and the temporal arrangements of police work (such as shift work, overtime, promotional process) have placed women at a disadvantage. Her analysis illustrates how initiatives, such as part-time and flex time, simultaneously “enable and disable women” because those who utilize such initiatives are perceived as not meeting the “ideal worker” category (Silvestri, 2018, p. 316). Thus, police work is organized in ways that disproportionately disadvantages women, yet organizational barriers do not tell the full story.

Police culture also plays an important role in shaping how gender inequality is created and maintained in policing. Yet, despite the explanatory power of police culture, the concept remains ‘complicated, contested and, at times, contradictory’ (Bacon, 2014, p. 103), with some arguing that researchers have fallen prey to simultaneously ‘oversimplifying’ and ‘over-intellectualizing’ the concept (Cockcroft, 2013, p. 11). Approaches to police culture have either treated culture as an undifferentiated phenomenon wherein officers ascribe to the same “complex values, beliefs, attitudes, expectations and norms” that shape their experiences and professional practices (Atkinson, 2017, p. 235), or they have ascribed police culture to an attitudinal characteristic (e.g., the ‘tough cop’ type) (see Ingram et al., 2013). Both approaches have been criticized “for being too broad, un-bounded, and loosely defined” - portraying culture as static and resistant to change (Ingram et al., 2013, p. 377, see also Chan, 1997).

Historically, much of the research on police culture characterizes it by ‘machismo’ (Manning, 1978; Reiner, 1992) and a ‘cult of masculinity’ (Brown, 1997; Fielding, 1994; Rabe-Hemp, 2009). But, Silvestri (2017) challenges conventional, singular accounts of masculinity and the over-emphasis on physicality and a ‘crime fighting mentality’ citing both as a deficit to policing and scholarship on policing and gender. Additionally, Campeau (2015; 2019) has sought to expand the explanatory power of police culture by drawing on discussions in the sociology of culture and organizations. Police culture, she argues, is “resourcefully used in practice” by police officers – such as through the use of myths, schemas, boundaries and scripts - to make sense of their experiences, practices and “social positioning in a hierarchy…” (Campeau, 2019, p. 70). Furthermore, the key to unpacking culture, she argues, is to “unveil when, where and how particular sets of cultural resources are put to work” (Campeau, 2015, p. 690). Thus, culture is shaped by the institutional structures within which it resides *and* the actions of officers.

Brotherhood is a prominent narrative in policing likening it to familial bonds (see du Pleissis et al., 2020). And, like many families, the *Brother*hood is rife with gender dynamics that shape experiences and inequities. As such, Connidis and McMullin’s (2002) articulation of structured ambivalence is particularly instructive. Focusing on family dynamics, they call for greater attention to the overarching structural and cultural elements that constrain, facilitate, and ultimately shape ambivalent behavior and meaning making. According to Connidis and McMullin (2002), “the contradictions and paradoxes of socially structured relations are reproduced in interpersonal relationships” (p. 559). They also argue that “ambivalence is created by the contradictions and paradoxes that are imbedded in sets of structured social relations (e.g., class, age, race. ethnicity, gender) through which opportunities, rights, and privileges are differentially distributed. Individuals experience ambivalence when social structural arrangements collide with their attempts to exercise agency when negotiating relationships…” (Connidis & McMullin, 2002, p. 565). As such, Connidis and McMullin’s (2002) concept of structured ambivalence can help to understand the complex and at times contradictory relationship individuals have with social phenomena and institutions.

By focusing on women’s experiences within policing, scholars have called attention to the complex negotiation women do to find a sense of acceptance and fit. Rabe-Hemp’s (2009) research has demonstrated how women’s ‘fit’ within policing is always precarious and demands constant negotiation. To negotiate fit, women engage in complex enactments of femininity *and* masculinity. For example, many women describe employing tactics of feminine deference such as self-selecting out of promotions and not resisting segregation into jobs within the department most often associated with women (for example, paperwork and dealing with crimes related to women and children) (Rabe-Hemp, 2009, p. 259). However, fit also requires expressions of masculinity as women are required to prove themselves by engaging in acts of aggression or violence and vying for higher ranking positions within their services (Rabe-Hemp, 2009). In this way, women are strategic about how they negotiate their gender to better their fit and advance their careers (see also Chan et al., 2010). While many women may find their ‘fit,’ the process of negotiating fit is not simply about their ability to *either* perform appropriate femininity *or* masculinity but rather navigate a strategic balancing act that both affirms and challenges traditional gendered expectations.

For Chan et al. (2010), women ‘do gender’ by affirming the notion that men and women are different (and should be treated as such), and ‘undoing gender’ when contesting gender-based discrimination in policing (p. 431). Women police often engage in a combination of doing and undoing gender wherein they acknowledge that men and women bring something distinct to the job; however, women should not be treated differently (Chan et al., 2010, p. 431). In this case, the doing gender model has moved away from *doing* masculinity, femininity, or a combination of both, and instead doing gender is centered on the notion of difference rather than gender per se.

Conversely, Garcia (2003) uses the doing gender model to understand the complexity of doing femininity in a hyper-masculine policing context. She finds, in some cases, doing a specific kind of femininity can facilitate access to certain jobs within policing; however, this process is regulated on the job and femininity can undermine women’s value as officers (Garcia, 2003, p. 339). Specifically, Garcia (2003) states, “if a woman acts too feminine, she is criticized for not being suitable for the job. However, if she acts too masculine, she is criticized for not acting like a woman” (p. 341). This bind illuminates not only the complex and competing tension women experience in managing gendered expectations, but it also an understanding that women will navigate this tension differently (for example see Morash & Haarr, 2012). Additionally, the complexity of gender negotiations also alludes to the possibility that the gendered substructure of policing works differently in different organizational contexts. Echoing Silvestri’s (2017) position, we must move beyond simplistic understandings of policing culture to better grapple with *how* gender matters across policing cultures. To do this, we put Campeau’s cultural framework into conversation with Connidis and McMullin’s (2002) structured ambivalence to demonstrate - the “dialectical tension between structured social relations and individual agency”– that shapes the processes whereby women police draw on the cultural resources of the Brotherhood, Boys’ Club, and Sisterhood in ways that shape their own sense of fit in policing (p. 564).

## Methods

Our theorizing for this paper is the result of a prolonged immersion into the study of women and policing in Canada over the past seven years. Specifically, it comes out of three different, yet interrelated, research projects that explored the experiences of women police officers in Canada regarding recruitment, retention, promotion and police cultures. Ninety-one participants [54 frontline officers (constable, detective and corporal); 25 management (detective sergeant, sergeant and staff sergeant), 7 senior management (inspector, deputy chief, chief) and 5 civilians (including special constables and an officer who resigned)] were drawn from thirteen police services across Canada; representing the experiences of municipal, provincial and federal police officers. All participants in the study were white presenting and ranged from one to thirty-two years of experience, with an average of twenty years in policing. The number of children for each participant ranged from 0–5 with an average of 2.

Half of the interviews were conducted by the post-doctoral scholar on the research team, while the other half were interviewed by the two faculty researchers. Throughout data collection, the research team met regularly to discuss early analytic insights and facilitate “progressive focusing” wherein we would refine our interview guide and research questions based on emerging analytic insights (Hammersley & Atkinson, 2007). As such, data collection and analysis were simultaneous, with one leading into and facilitating the other. This approach to data collection allowed us to continually clarify topics and engage in theoretical sampling and saturation (Charmaz, 2006). These team meetings also presented an opportunity to facilitate ‘researcher triangulation’ (Hammersley & Atkinson, 2007).

Interviews were digitally recorded and transcribed verbatim. All data were coded and analyzed by the post-doctoral researcher drawing on Charmaz’s constructivist grounded theorizing (2006). Constructivist grounded theorizing prioritizes the experiences of the participants while allowing researchers to be reflexive and draw on relevant pre-existing theoretical concepts and insights. Using qualitative software (NVivo 11), we began by identifying relevant themes (e.g., ‘Brotherhood’, ‘Boys Club’, ‘Sisterhood’) and “sensitizing concepts” (Blumer, 1969), such as police cultures. After identifying recurrent themes, we used writing as an analytic device to make sense of the themes and draw connections and distinctions among and between them (Charmaz, 2006). Through our prolonged immersion in the data and our engagement with analytic memos, we began to make sense of the way women police utilized cultural resources, such as Brotherhood, Sisterhood and the Boys’ Club to navigate their ‘fit’ and inclusion in the hierarchal institution.

### Using Cultural Resources to Navigate ‘Fit’ in Policing

I went to a meeting the other day, there was twenty-two people there, there’s three women. … I routinely go to meetings - I went to one…and the people were saying, you must be [name]. I’m like wow, you must be psychic, that’s great. And then I sit down at the table and go, oh wow, now I know why everybody knows who I am, I’m the only woman here. And that happens to me all the time. (Della)

Policing is gendered in complex and contradictory ways as evidenced by the way women use cultural resources including the Brotherhood, Boys’ Club, and Sisterhood to make sense of their gendered experiences and ‘fit.’ In reference to the Brotherhood, women engage in processes of gender neutralization wherein the distinctions between men and women become implicit. Conversely, the cultural resources of the Boys’ Club and Sisterhood were deeply gendered, both framed as a barrier to women’s fit. In what follows, we begin by unpacking the strategies of neutralization found in navigating the Brotherhood of policing.

### Gender Neutralization and the Brotherhood

Much of the research available on policing has utilized the narrative of the ‘family’–e.g., “Blue Family” (du Pleissis et al., 2020) and “Brotherhood” (Campeau, 2019)–to refer to a ‘sense of belonging and service as a family’ that distinguishes policing from other realms of work (du Pleissis et al., 2020, p. 5). This is also the case with the women we spoke with. As Darlene explains, “we’re all blue, we’re blue together, this is our family.” Here Darlene intertwines ‘blueness’—a colour symbolic of policing—with familial ties to illuminate the prominence of intimacy and solidarity in the familial relationship. Yet, surprisingly absent from most of the reflections on the Brotherhood was an explicit appreciation of the gender-coded language and implications thereof.

Whereas the *Brother*hood overtly refers to the camaraderie and solidarity among *brothers*, most women describe the Brotherhood in ways that obfuscate the gender-coded language. Through their articulations of the Brotherhood, women engage in a process of neutralization of gendered connotations while emphasizing a sense of belonging, protection, and camaraderie. As Judy states,

So, I think you have that crazy camaraderie, this brotherhood, that you do feel so protected, and instinctively you want to protect each other, you all do, and you look out for each other, and when there’s somebody that comes in, and, you know, they’re trying hard and they’re doing their job, and they’re struggling, people look out for you…yeah there’s definitely a brotherhood.

In this way, women utilize the Brotherhood to describe the protection, solidarity, and cohesion afforded to them within the institution of policing. Those that are afforded inclusion in the Brotherhood are spoken of in gender-neutral terms. Even when women noticed the gender coded language of the Brotherhood they would neutralize that coding by distancing their own interpretation from one that foregrounds the gendered subtext of the *Brother*hood by positioning it as a concept that, as Kate says, is available to “any uniform-wearing person.” As Darlene explains further,

I don’t personally think that it has anything to do with whether or not you’re a girl or a guy or anything, it just has to do with the fact that you’re here, and you’re having to do this job …and this is going to be your life, and we’re all here together to deal with that.

Our participants’ use of the ‘Brotherhood’ provides greater insight into the complex ways in which women navigate their gender and ‘fit’ in policing. For example, Kate expands on her understanding of the brotherhood,

Where I think brotherhood, there’s something to be said about a person who works in uniform and being together as a brotherhood*.* I have never felt excluded because of being female, from doing any activities with the guys. I mean they’ve had ‘guys’ weekends’ where they’re going hunting, fill your boots, I don’t even want to come, you know what I mean?

Here we see a shift in the way this officer understands the brotherhood (inclusive of all those in uniform) and her own exclusion from “guys’ weekends.” While she may have no interest in attending these “guys’ weekends,” by establishing them as such, she highlights the central role of gender as a boundary between work and play, fit and exclusion. In this way, gender distinctions in camaraderie building and familial support is framed in terms *outside* of police work. As such, demarcating the ‘gender-neutral’ brotherhood bond at work, one wherein this officer is included, from the gendered bonding outside of work, is a means of neutralizing the gender-coding of the brotherhood as a concept in policing.

In other cases, the gender-coded language of the brotherhood is neutralized, but ironically the process of neutralization simultaneously reifies the centrality of men. As Veronica, states,

I don’t look at it though as a brotherhood or a sisterhood, I really look at it as, we’re all in this together, yeah. And that may be easier because my husband’s a police officer, my dad was, so*,* I tend to relate to men a little easier at work, maybe because of that, I don’t know.

By addressing the gender-coded language of the Brotherhood and inserting attention to a potential Sisterhood, Veronica attempts to minimize the significance of gender in relation to ‘fit’ within policing. However, the next sentence effectively affirms the centrality of men in policing when she establishes her own ability to fit into a male dominated space. Similarly, Marissa explains how she has always ‘fit’ in the Brotherhood because:

I’ve been a Tomboy my whole life, I love to joke, I’ll laugh at the crudest, rudest jokes, so, I fit into that and I’m okay–in fact I like it. I feel the most comfortable in this organization when I’m working with a group of guys like that.

As described above, both officers cite their ability to relate to men as a vehicle for their own inclusion and fit in policing. Additionally, while the Brotherhood is outwardly understood as gender-neutral and reminiscent of a family, Marissa’s fit relies upon her ability to perform her own version of stereotypical masculinity. By articulating her own distance from normative femininity via the “Tomboy” label and her own celebration of “crude” and “rude” behaviors, she illuminates the implicit coding of appropriate gender in her own police culture. While many women attempt to neutralize the role of gender in the Brotherhood, this process becomes complicated when women discuss challenges and dissent *within* the Brotherhood.

Whereas all officers associated the Brotherhood with solidarity and protection, some also articulated clear boundaries and an informal code of conduct that officers are required to maintain their inclusion within the Brotherhood. As Macey describes, the Brotherhood provides officers with “[t]he support, the standards, the family, the ‘I have your back’, but it is also, ‘*don’t snitch*’, ostracize people that do, but again, *don’t forget about that loyalty piece*” (*emphasis added*). In this way, one’s ability to fit within the brotherhood depends in part on one’s ability to toe the line.

During our conversations with women, it became clear that many of them understood dissent within the Brotherhood as a gendered phenomenon. We quote Jessica at length here,

So, you’re a member of the Brotherhood, *unless you challenge the men*. So, I really do think that, I think that the women who challenge men, are immediately ostracized. So, when you have those women who cry harassment, and I’m going to say ‘cry’, cause that’s *their* language, ‘cry harassment’, complain about bias, as soon as you challenge that, you’re ostracized…and you’re also ostracized by the women, don’t get me wrong…you thought you would be treated fairly on the merits of your complaints. You absolutely will not be. You will not be. It’s *you* against *them*. It’s *them*. So you’re challenging the integrity of that Brotherhood, when you challenge-and I don’t mean individually, if somebody does something wrong to you, blatantly wrong to you, you might get some support, but when you start to challenge behaviors, which may manifest themselves in an action towards a woman, the *good Ol’ Boys club* or those sorts of things, or discrimination, forget it. You’re out. You’re absolutely out. And, I mean I’ve never had that experience, but I think we, uh, edit our behavior, or edit stuff, because we know…intolerable behavior in today’s day and age, was something I absolutely had to tolerate. And I knew I had to tolerate it. Absolutely. Because, it would have been me against them. *All of them*.

Curiously, Jessica initially frames the Brotherhood in relatively gender-neutral terms and in doing so she also establishes her own fit within. However, when discussing the nuances of that fit, gender emerges as a central characteristic to the Brotherhood itself. She does not state that challenging the Brotherhood is problematic per se, instead, she clearly establishes that any challenge to the “behaviours” or “discrimination,” specifically “challenge to the men” results in the immediate ousting of women officers. Jessica also reaffirms her own fit within the Brotherhood when she isolates herself from these gendered exclusions, she states, “I’ve never had that experience.” In this way, she effectively reminds us that women *do* belong in the Brotherhood; however, belonging requires tolerating “intolerable behaviour” and regulating (‘editing’) one’s behaviour. We also see the slippery relationship between the ‘Brotherhood’—a presumably gender-neutral concept—and the ‘Boys’ Club’—a deeply gendered and discriminatory phenomenon (one we explore in greater detail in the following section).

Many women position the Brotherhood as a relatively open space wherein all are welcome; however, some women *choose* not to participate. Thus, the inability for individual women to fit within the Brotherhood is framed as a decision that women themselves make rather than coerced to make. Consider Tiffany’s perspective: “I find that the females who just can’t get on board and deal with it often pull themselves away, and you know, don’t have that same relationship with the guys, but it is a Brotherhood for sure, and you know I love it.” For Tiffany, women who are unable or unwilling to “get on board” or “deal with” the norms of the Brotherhood are not excluded but rather are self-selecting out of their possible inclusion. Further, by positioning herself within the bounds of the Brotherhood, one that has positive connotations to her, Tiffany makes it less about women’s lack of fit and more about ‘those women’ and their inability or unwillingness to fit. As such, the gendered underpinnings of the Brotherhood are framed in gender-neutral terms (Tiffany’s ‘fit’ affirms this). In this way, the attention shifts away from larger questions about cultural and structural inequality embedded within the Brotherhood and instead toward the decisions that individual women make about their careers. The framing of the Brotherhood as gender-neutral is a neutralization process, whereby the gender coded expectations require for ‘fit’ remain implicit and intact.

### Emphasizing Gender in the Boys’ Club and the Sisterhood

While the gendered substructure of the Brotherhood is often neutralized in our conversations with women police, cultural resources such as the ‘Boys’ Club’ and ‘Sisterhood’ are explicitly gendered. As a cultural reality with a rich history of critique, the Boys’ Club for many women signifies a subset of policing that creates barriers to fit, most notably to their advancement through the ranks. Conversely, most women push back against the very existence of a Sisterhood. Certainly some women note the potential benefits of solidarity among women in policing, however, the majority of women we spoke with understand the Sisterhood to be a potential threat to their own inclusion in the dominant Brotherhood culture.

### The Boys’ Club

Research available on police culture has often referenced the ‘Boys’ Club’ (Langan et al., 2019) and ‘Old Boys’ Club’ (Campeau, 2019) when describing the masculine ethos of the occupational culture of policing (Brown & Silvestri, 2020). Unlike the neutralization of gender as it relates to the Brotherhood, the Boys’ Club was used by women police to describe and make sense of exclusionary practices and relationships. As Kim explains, while the ‘blue line’ extends across all members of the service,

[T]here are still *some old school officers* out there, supervisors, but even some guys that have just been on the road for thirty years, that just still don’t have as much respect for female officers, may not have as much respect for the fact that I may have more of a tendency to talk to somebody longer, so that we don’t have to fight, as opposed to them just kind of jumping in.

For Kim, and many others, the Boys’ Club was generational and informed by an outdated mentality that reinforced a masculine ethos rooted in physicality and the valorization of traditional crime fighting. As Rachel delineates,

I’m walking the beat and there’s a pile of guys in the front. So, I get in the middle and I’m trying to get them in, you know. So, fists are swinging and everything else, I get the one guy out, I ended up cuffing him, and I’m looking, where’s my backup, where, you know, where’s anybody else, there’s a crowd of people standing around. [I’ve] got nowhere to put this guy, [so I] ended up cuffing him to a tree to go over and help the others. All of a sudden when everything’s done, I hear this [claps] and I look down the road. They were watching, the *guys* were watching me. And I was mad. I was like, ‘what the fuck was that, you know, what are you doing?’ And, ‘oh just want to make sure you can handle yourself, be *one of us*.’ But it’s almost like you have to prove yourself, if you want to be part of that Old Boys' Club.

As described above, the Old Boys’ Club is used to make sense of a time where her ‘brothers’ did not provide her support and protection, but instead required her to demonstrate her ‘fit’ and ability ‘to be one of us’. In this way, the Boys’ Club is deeply entrenched in a traditional crime fighting mentality that requires women to get in there and ‘put your hands on people,’ make big arrests, and catch bad guys to earn their place. As Olivia notes,

I feel like-and other women feel this too…we are sort of seen as not so great until we *prove* ourselves otherwise. Whereas when a guy comes in, he’s seen as good until he proves himself to be not good. *And that has always been a very big challenge*, and … that’s why *I’ve always never been afraid of physical confrontation*, or, you know, engaging in any sort of, you know, *chase* or whatever, because, and *it’s always been really important to me to show that.* (emphasis added)

For Olivia the need to demonstrate her willingness to be physical and to engage in the ‘chase’ has been important for her to demonstrate her ‘fit’ in policing. For some women, like Susan, the pressure to prove oneself has left them to “live in this box of having to be cool macho” and to “mold [themselves] to become that in order to do the job.”.

For many women, the Boys’ Club was also used to make sense of the *limited* advancement and inclusion of women in the “inner circle” (Saundra). As Susan and Rachel explain,

[I]f you look at our hierarchy and the number of women in positions of power in our organization, I don’t think I need to say anything else. Like truly, you’ve got one, we had a [high rank] who was a female, but you know she was married to a cop, her dad was a cop, … her dad was [a top ranking officer] at one point, and she was married to a cop, the *nepotism* within it is… they talk about inclusion and diversity, and how they want that to be the way our police services run, and yet *they all belong to this club that excludes women,* what the fuck, like are you kidding me? And so, *how do you fight that,* … *so I will never, ever be able to get into their little circle of the Boys’ Club in any way shape or form.* (Susan, emphasis added)

If you *were lucky enough*, at that time, they sat down as a group of the Old Boys and said, ‘yeah, she gets an interview, no she doesn’t, or he gets an interview, no he doesn’t’. … there was an Old Boys' Club, and *they had control of everything*, and it would never change. (Rachel)

For both Susan and Rachel, the Boys’ Club is a powerful entity (“they had control of everything”) that is maintained through nepotism and blatant favouritism. Some women who are willing to strategically ‘play the game’ and ‘toe the line,’ can find a way into the inner circle. Yet, to strategically ‘play the game’, many women felt that they had to distance themselves from each other and “sort of join on the bandwagon with the men, and not be as friendly to the other women, so that the acceptance happens” (Connie). In this way, the Boys’ Club, unlike the Brotherhood, is understood in explicitly gendered terms and most often recognized as an explicit barrier to fit for women in policing.

### The Sisterhood

The notion of a Sisterhood, like the Boys’ Club, is deeply gendered. While all women note the existence of a Brotherhood and Boys’ Club in policing, almost all refute the existence of a Sisterhood; however, explanations for why there is no Sisterhood varied. For some, a Sisterhood, although regarded as potentially helpful, did not exist because of broader systemic material, symbolic, and institutional conditions that foster competition and divisiveness among women police. For these women, Sisterhood was perceived as a threat to the status quo. As both Susan and Connie explain,

I think too many of the women are trying to get into the Brotherhood, and I don’t think we, I try too, I don’t think we support each other enough, I really don’t. I think if you’re seen supporting, um, if you’re seen supporting other women, you’re looked at in a negative light by the guys. And so, I don’t think it happens enough, for that reason only. (Susan)

I just think there’s a lot of pressure on women in general, in policing, to sort of measure up, gaining the respect of the men, and, I don’t know. And there’s just, I think that pressure sort of complicates things as far as women bonding together, and standing up for each other, and backing each other up, and supporting each other as opposed to, you know, joining with the masses … (Connie)

While framed as gender-neutral at the onset, for both Susan and Connie, and many of the other women, the gendered nature of the *Sister*hood is a catalyst to re-open discussions about the role of gender in the Brotherhood. Unfortunately, the discussion shifts the focus away from the potential benefits of a Sisterhood and toward the maintenance of a gendered status quo that prioritizes the culture of masculine dominance. The mere mention of a possible Sisterhood renders the gendered nature of the Brotherhood visible. Further, the Sisterhood signifies threat to the status quo and thus, a barrier to inclusion.^3^

For others, the lack of a Sisterhood within policing reflects the competition between women. As Kate muses, “I wish women at work were more supportive of each other, and I’m probably just as guilty as they are, but I don’t know if we treat each other as rivals.” Deana also recognizes the gendered nature of competition when she states, “We eat our own around here. Every female’s jealous of every other female, it’s insane here. We do not support each other at all… you won’t see too many females here that are friends with other female cops.” Competition is central to the discussions about why no clear Sisterhood exists. Not only is competition required to successfully navigate ‘fit’ but a drive for competition is part of how women express their desire to advance up the ranks. As Linda states,

Women can be real bitchy with each other, and cutthroat. Because it’s almost like, I want to be the top bitch. *I want to be the one in charge of the shift*. It’s really weird sounding, when I say it out loud, but I know that exists.

Jessica echoes, “I really thought that police women would stick together and help each other out, but it was a minority, I was really surprised by the cattiness, and just overall backstabbing that went on with female officers…it’s so competitive, extremely competitive amongst female officers.”

For many women, the competition that erodes the potential for Sisterhood lies in the male-dominance of policing. According to Fatima,

I think that we’re all A-type personalities, I think every police officer is an A-type personality, I don’t know I think maybe it’s competitive to be the top female in a male-dominant job…I mean, you’ll get the few girls that will have a sisterhood, *but that seems to stir it*. I don’t bother with that. Like I have a group of four or five close girlfriends, but we’re very tomboyish, and we just don’t want to deal with that.

While Fatima acknowledges her own association with a small group of women, she positions herself and those women outside the bounds of a Sisterhood. Interestingly, gender itself is used to create this boundary. In this way, the concept of a Sisterhood is another resource women use to navigate their ‘fit’ albeit in different ways than the Brotherhood. The women who ‘stir it’ are those who challenge the male-dominance of policing, while the ‘tomboy’ marker is a means of distinguishing oneself (and her close friends) from those women who stir the pot of male-dominance. For Fatima and others like her, using ‘tomboy’ as a marker of female masculinity, she effectively offsets any potential threat to the dominant culture associated with a collective of women.

While the concept of a Sisterhood is understood to be an impediment to the broader push for a gender-neutral police culture, others point to the structural elements of policing that erode any potential for solidarity among women. Consider both Judy and Tara’s comments:

Women feel directly in competition with other women because each team, let’s say of Drugs or Intel, it’s not a balance of five girls and five guys, it’s generally ten guys and one girl, or four guys and one girl....So you’re in competition with the women, you’ve got to be the best woman to get that position, because you know that if you’re the best of the women, you’re going to get that position, right, because there’s always going to be a spot for a woman… And so you’re directly head-to-head with them, which is what breeds the cattiness between the women. (Judy)

There was, a woman spot in the units…I called it “The token ovary spot.” So, when that spot became open, it was generally filled by another woman. So, because of that, we fought each other. This culture was created where in order for us to move forward, we were at each other. Then it was, so then that, added credence to the myth that too many women in one place somehow was? Like you know? Quite frankly, we have to look inside ourselves, we were responsible for some of that. You know? I will take responsibility for not saying “You, not me this time” even if there was one spot, we did not. (Tara)

In both cases, women bring attention to the limited structural opportunities available to women because of the gendered competition. While there is likely no official mandate for only one woman to be successful in competing for a position, the likelihood that more than one woman will advance in an open competition is something women are aware of. Interestingly, rather than going further and questioning the merit of informal concepts like the “token ovary spot”, she downloads the responsibility onto individual women like herself and others who did not step aside to let another (one) woman in.

On a few notable occasions, officers identified the barriers to Sisterhood in the systemic operation of policing. As Jan explains,

If there are three jobs posted that people are competing for and five of those people competing for those two or three jobs are women, those five women who are competing for that job, they know damn well that those three positions are not going to be filled by women. So immediately, their competition is not me against all the other competitors, it’s me against those other women…so *they set us up to compete against each other* and be catty and undermine each other. I mean people do that in competition anyways, but *women are worse, because they know you’re not competing fairly*.

Like the previous example, Jan identifies the competition as a barrier to Sisterhood. In this way, competition is not a result of individual women or stereotypical behavior, it is the result of the institutional structure and organizational culture.

While some women recognized the potentially positive aspects of a Sisterhood, there were just as many women who identified the Sisterhood as a negative concept. Unlike the ‘gender-neutral’ concept of Brotherhood, the critics framed Sisterhood as a gendered, and therefore, negative concept. These arguments typically drew upon a logic that identifying ‘difference’ is a hindrance to inclusion. Thus, women saw Sisterhood as a means of drawing attention to ‘difference.’ As Carol exclaims, “I find that whole business very—like we are taking a step backwards, I am not part of the women’s one. I don’t really understand how on one hand you can want equality but on the other hand segregate this little group. It seems competing to me.” Similarly, Joyce questions, “Why do you need that? Why don’t you just, why don’t we just pick the best police officers and sort of raise them up?” Or as Marissa says, “I’ll have zero part of that, because forever and ever, as long as we keep ourselves separate…we’ll continue to keep the separation…*I’m not part of any female undercurrent*…” (emphasis added). In each case, Sisterhood is positioned outside the realm of fit, as separate to the presumably gender-neutral Brotherhood. In the case of Marissa, calling out ‘difference’ is in some way associated with an “undercurrent”, or rebellion against the supposed gender-neutral status quo.

## Discussion

Social movements and citizens in Canada and elsewhere are calling for widespread police reform. Some argue that ensuring police organizations better reflect the communities they serve will effectively erode the rigid division between ‘us’ and ‘them’ creating more space for progressive changes. However, as our research demonstrates, inclusion and diversity alone are not enough to change an organizational culture or larger structure. There are deeply embedded realities that inform and constrain individual agency. By examining how women utilize and reject three prominent cultural resources of the Brotherhood, Boys’ Club and Sisterhood, we provide a nuanced account of how gender is strategically navigated within policing in ways that demonstrate a structured ambivalence about the role of gender in shaping experiences of policing. We agree with Campeau (2019) that police culture is not a set of internalized individual beliefs or attitudinal typologies, but instead involves powerful “coherent orientations to the dilemmas institutional life pose” (p. 763). Our research clearly demonstrates the nuanced variations in how women interpreted the cultural meaning of the Brotherhood, Boys’ Club, and Sisterhood. Through strategies that both neutralize and emphasise the importance of gender, women demonstrate the complex relationship between structure, culture, and on-the-ground practices.

Participants are heavily invested in the discourse of family most notably as it applies to the narrative of a Brotherhood. This use of the Brotherhood signifies its connection to a symbolic boundary of protection, camaraderie, and solidarity around *all* uniform wearing police officers - not just men. Interestingly, the Brotherhood was used to reinforce a symbolic boundary around policing–the ‘family’–against those *outside* of and *external* to it. For our participants, the Brotherhood, was necessary to protect officers against the dangers (potential or real) associated with dealing with outsiders. This symbolic boundary around policing, however, reinforces specific values and practices, such as ‘don’t snitch’ and ‘toe the line,’ values that effectively maintain an organizational status quo. The way in which this cultural resource is used allows women to neutralize the gender-coded connotations of the *Brother*hood itself thereby enabling their ‘fit’ within the hierarchical and gendered institution. Yet, the gendered dynamics of the Brotherhood are not entirely neutral. In establishing the requirements of fit—toeing the line, not challenging the status quo—the gendered elements of the Brotherhood become clear. For women to fit, they must be able to ‘deal with’ the status quo, one that is clearly steeped in a specific kind of masculinity and gender hierarchy. In this way, the relationships women have to the Brotherhood are complex and nuanced.

Conversely, women draw upon the cultural resources of the Boys’ Club and Sisterhood when making sense of and navigating gendered challenges that exist *within* policing. For example, women would draw on the cultural resource of the Boys’ Club to make sense of discriminatory practises within their service (such as nepotism and blatant favouritism) and to carefully *navigate* their fit within its gendered hierarchy. Women resourcefully used the cultural concept of the Boys’ Club and its elements (i.e. abiding by the hegemonically masculine ethos and adopting a crime fighting mentality) to make sense of structural realities (such as the lack of institutional opportunities for women to advance in units–‘token Ovary spot’) that constrain and facilitate their work.

Although many of the women recognize the historic and material conditions that frame their experiences within policing–such as the low number of women in policing and only one woman per specialized unit–their everyday actions did not necessarily acknowledge these macro-level conditions. In this way, women’s understandings of gender are informed by a wider structured ambivalence (Connidis & McMullin, 2002). For example, their acceptance of the gender-neutral discourses of the Brotherhood and their active rejection of the gender specific Sisterhood allowed women to navigate the institutional hierarchy and their ‘fit.’ The investment in, and rejection of, gendered discourses, we argue, is *strategic*. To successfully ‘play the game’ women must navigate the hierarchical structure and deploy cultural resources–such as a masculine ethos and crime fighting mentality; however, through their adoption and use of these cultural resources they reproduce the very beliefs and practices that perpetuate the gendered nature of this work. Our findings make evident the strength of the occupational culture-and the way in which it is shared and reinforced through both hierarchical and horizontal relations in policing. As such, we illustrate why it is difficult for women police to build solidarity (or Sisterhood) when they are actively engaging in strategies of fit that, paradoxically, reinforce subtle gendered boundaries.

Our analysis seeks to advance discussions and theorizing on gender and police culture. By putting cultural elements in conversation with the concept of structured ambivalence we move beyond simplistic and deterministic definitions of police culture as hypermasculine, to provide a nuanced analysis that attends to the complexity and multiplicity of gender representations and negotiations within policing. Through the use of ‘structured ambivalence’ we illustrate how women police draw on cultural resources (such as the Brotherhood, Sisterhood and Boys’ Club) to navigate their fit. As such, our analysis, similar to Brown and Silvestri (2020), calls for a gender-centred cultural change. Further, the use of structured ambivalence demonstrates both the potential for agency and the constraints on that agency when drawing on cultural resources. Not only is change possible, but it is necessary. Structured ambivalence, therefore, allows for change because contending with ambivalence requires some sort of action–whether it is to resign oneself to the gendered hierarchy (i.e., not participate in the promotional process), ‘play the game,’ or fight against it. As Connidis and McMullin (2002) note, “…when substantial numbers of individuals who share a similar position attempt to negotiate the ambivalence created by current structural arrangements, there is the potential for change” (p. 565). The approach taken and the possibilities for change, however, are shaped by the variability of available resources.

Our study contained variation in rank, family structure, size of service, and in some cases sexuality; however, these intersections did not directly inform our analysis. As the significant body of scholarship on intersectionality can attest, gender negotiations are shaped by race (among other social categories). Further, because race and gender are inextricable, race (whiteness) undoubtedly shaped the way women navigated gender resources in our study; however, a thorough analysis of race was not central to our analytical framework. We see this as an important area for future study. Future research would also benefit from examining how rank, social identity, and sexual orientation shape access to, and negotiation of cultural resources.

## Conclusion

Research on women and policing has provided helpful insights into the gendered nature of policing and the role culture and cultural resources play within. Although police services are facing increased media and government pressures to engage in diversity reforms, our analysis shows that the simple inclusion of more women within the service will not in and of itself create change. Rather, organizational reform needs to be addressed at both the policy and practice level and requires an awareness of where the cultural barriers and opportunities for change exist. By examining how women utilize cultural resources, such as the Brotherhood, Boys’ Club and Sisterhood, to make sense of their experiences and navigate their ‘fit’ within the service we uncover how complex understandings of gender are shaped by larger gender structures.

## Notes

1. Senior officers include those officers at the lieutenant or higher, ‘such as chiefs, deputy chiefs, staff superintendents, superintendents, staff inspectors, inspectors, lieutenants, and other equivalent ranks’ (Statistics Canada, 2022).

2. We recognize that we cannot do justice to the historical and ongoing debates within feminist literature and activism spaces about what this term connotes in the space allotted here. As such, we are using a popular understanding of ‘Sisterhood’ to signify the potential shared sense of solidarity among all women as understood by the participants.

3. For more on the perceived threat women pose to police culture see, Brown et al., 2019; Chan et al., 2010
